# Perspectives on Implementing Environmentally Sustainable Practices in Cataract Surgeries: Interviews of Administrative and Frontline Healthcare Workers

**DOI:** 10.21203/rs.3.rs-7482318/v1

**Published:** 2025-10-06

**Authors:** Daniel Parra, Brooke Sherry, Emma Pak, Lauren Taylor, Erin S. Rogers, Sarah E. Hochman, Maria de Los Angeles Ramos Cadena, Joel S. Schuman, Christina R. Prescott, Cassandra L. Thiel

**Affiliations:** The Warren Alpert Medical School of Brown University; New York University Langone Health; New York University; New York University Langone Health; New York University Langone Health; New York University Langone Health; New York University Langone Health; Wills Eye Hospital; New York University Langone Health; New York University Langone Health

**Keywords:** Environmental sustainability, Change implementation, Health system growth, Healthcare policy, Frontline healthcare workers, Administrative healthcare workers

## Abstract

**Background:**

Healthcare is responsible for 8.5% of greenhouse gas emission in the United States. Physicians are becoming increasingly concerned about the climate crisis, particularly in the field of ophthalmology where there is a growing body of literature related to sustainability. Although emissions of cataracts surgery, one of the most performed surgical procedures in the world, have been quantified, modifications to practice have yet to be made. This study aims to uplift the perspectives of a diverse set of healthcare workers on implementing environmentally sustainable practices in the cataract surgery setting.

**Methods:**

15 semi-structured interviews were conducted with professionals working in various direct patient care or administrative roles at a large health center to gain insight on implementing a variety of sustainability initiatives. We focused on initiatives related to supply reduction, reusable supplies, multi-dosing pharmaceuticals, and health system process and policy shifts.

**Results:**

Participants most frequently identified infection prevention and control concerns as a primary barrier to implementation. Additionally, the infection prevention and control department was most often cited as a key stakeholder in implementation. However, participants from this department did not share these same concerns. Additionally, participants most often cited that these initiatives would be successfully implemented from the bottom up, meaning driven by those providing direct patient care.

**Conclusions:**

Themes generated from the collection of responses underscore a broader discussion of disconnect between policy and practice in healthcare as a barrier to implementation of these initiatives and an opportunity in harnessing bottom up change to implement sustainable practices in a growing healthcare system.

## Background

Addressing the ongoing climate crisis is of increasing concern to the general public and to healthcare professionals (1). Related to eye care, climate change will likely increase the prevalence of ocular trauma secondary to extreme weather events, vector-borne diseases such as trachoma (2–4), onchocerciasis (5, 6), and eye pathology related to excessive heat, ultraviolet radiation, ozone, and other environmental pollutants exposure (7–14). The healthcare industry emits about 5% of the world’s climate-changing greenhouse gasses (GHGs) (15). The proportion is higher in the United States, where approximately 8.5% of the total GHGs originate from the healthcare sector (16, 17).

Sustainability efforts within the healthcare sector have been expanding, with ophthalmologists leading their medical peers in the number of studies produced assessing their GHG impacts and potential interventions. In high-income countries, a majority of emissions from cataract extraction originate in the manufacturing and distribution of single-use supplies (18). There is substantial variability in waste generation and emissions from cataract surgery in different countries, highlighting an opportunity where environmentally conscious change is possible (19–21). Endophthalmitis studies, a common complication of cataracts surgery, at Aravind Eye Care System in India raise the possibility that many of the costly and wasteful infection control protocols utilized by operating rooms in the United States could be unnecessary (22–24).

Surgeons and clinicians, in ophthalmology and beyond, are gaining interest in sustainability and looking toward more responsible practices (24). A pre-pandemic survey of ophthalmic surgeons and nurses showed overwhelming support for sustainability interventions such as reuse of supplies and multi-dosing of medications (25), with those in Ambulatory Surgical Centers (ASCs) more likely to state that they are already engaged in these practices (26). As a result of the survey, the American Academy of Ophthalmology (AAO) atnd American Society of Cataract & Refractive Surgery (ASCRS) joined the in 2020, providing their members access to educational and advocacy resources implement these sustainability interventions (27).

Despite increasing knowledge and a growing desire for action, many healthcare practices in the US remain unchanged, particularly in large health systems, hospital-affiliated outpatient surgery centers, and academic medical centers. Previous studies neglect the perspectives of allied healthcare workers, focusing largely on the surgeon’s perspective. While the surgeon is typically a powerful role in a healthcare facility, many other stakeholders also influence policies, practices, and procedures. As interest in climate action grows, particularly in the healthcare space, we aim to better understand the mechanisms by which this climate-forward change can be most effectively implemented.

## Methods

This study was conducted at a large academic medical center comprising of six inpatient facilities and over 300 ambulatory healthcare centers. In the fiscal year 2019, this health system had over 4,000 physicians and 7,000 nurses who supported 90,000 admissions and 7 million outpatient visits. In 2022, the Ophthalmology Department, with 161 faculty and 63 surgeons, performed over 5,400 procedures, of which 2,900 were cataract surgeries. A majority were performed in the Eye Center’s outpatient ambulatory care center.

In this study, we developed a semi-structured interview guide to gather information on healthcare professional’s concerns and vision for implementation pathways for certain intervention domains in ophthalmology. The initial section of the interview guide included questions about the interviewee’s role, degrees, and length of employment at the study’s location. The remainder of the guide focused on interviewee thoughts related to specific interventions that, if used, have the potential to reduce healthcare related GHG emissions.

The intervention domains include eliminating or reducing supplies, reusable supplies, pharmaceutical waste reduction, and process change, shown in Table 1. These specific domains were chosen based on previous literature on sustainability in ophthalmology and other healthcare settings (24). Within each domain, 3 to 5 specific interventions were chosen based on appropriateness for the study location. For example, within ‘eliminating or reducing supplies’, we asked about interviewees’ perspective on “replacing a full body drape with a face-only drape.” With those domains and interventions in mind, the interview guide included questions about concerns and barriers to implementing the intervention, data gaps and needs, individuals or groups needed to support each intervention, and potential steps to take in order to implement each intervention in the respective domains.

Finally, the guide asked interviewees for their general thoughts on potential stakeholders within and outside the health system who might offer additional valuable perspective. Two open-ended questions were asked – one for the interviewee to give general thoughts and feedback, and the second for the interviewee to recommend others whom we should interview for the study.

Given the complexities of health systems and the multidisciplinary nature of sustainability interventions, we purposefully selected a broad spectrum of professionals – both within the Ophthalmology Department and the larger institution. Interviewees were identified with a convenience sampling approach, based on their role within the health system, previous collaboration with other sustainability projects and studies within the system, and through word-of-mouth suggestions at the end of each interview. Our study did not require an ethical board review because it does not directly involve human subjects. Written consent was obtained prior to the interview, and verbal consent was obtained once again during the interviews for all participants.

### Data Collection and Coding

Interviews were conducted virtually with each individual interviewee, invited via email, by a single interviewer from the research team. Interviewers came from diverse backgrounds and were graduate students and research assistants affiliated with the senior author’s research group with varying levels of experience in qualitative research and healthcare sustainability. The interviews lasted approximately an hour and were recorded with the verbal consent of each participant and then transcribed using automatic transcription software provided as a part of the interview tool. Following the interviews, a member of the study team reviewed the recording for semantic and grammatical errors to ensure accuracy of the transcript. Transcripts were not returned to participants for review.

We used deductive and inductive approaches to coding the data. An initial codebook was developed based on a previous survey of cataract surgeons, conducted by this research group (25). The initial codebook was developed on a digital spreadsheet and tested by 3 members of the research team (2 primary coders and 1 conflict resolver) using the first 3 interview transcriptions, with coding consensus between reviewers ranging from 89%−93% in the first three transcripts. Following this initial independent coding, the research team reviewed coding decisions, discussed deviations in coding practices between team members, and added additional codes to the codebook. Subsequent coding consensus ranged from 95–99% between the coders. The final codebook was used by 2 independent coders from the research team, and their coded data was consolidated by 3rd research team member.

### Analyzing data and follow up interview

Thematic analysis was used to generate conclusions and explanations for our research question regarding barriers and facilitators to implementation of sustainability initiatives in the ophthalmology setting. Specifically, interview response consolidation allowed for identification of themes and subthemes of commonly perceived barriers, data needs for implementation of sustainability initiatives in the ophthalmology setting, and key stakeholders perceived as having influence over the implementation of these initiatives.

Based on these identified themes, we conducted a follow up unstructured interview with a representative from the Department of Infection Prevention and Control at the case study health system to gain further insight into themes that emerged across participants’ interviews and to make specific recommendations on successful implementation of sustainability initiatives in ophthalmology.

## Results

### Overview and Interview Demographics

Our interview sample consisted of 15 professionals working across the case study’s Eye Center and health system, with no interview requests denied. Interviewee perspectives were informed by their roles, which included Nurses (2), Nurse Manager (1), Scrub Technician (1), Surgeons (4), Pharmacist (1), Operations (1), Regulatory (1), Finance (1), Hospital Epidemiologist (1), Sustainability (1), Ophthalmology Department Management (1), and Health System Administration (1) personnel. Of the interview sample, 8 of the 15 participants worked in clinical roles providing direct patient care, including scrub technician, nurses and surgeons, and 7 of the 15 participants worked in logistical and administrative system roles. Amongst these professionals the median years of experience working in this specific health system was 10.5 years. Interviewees recommended 3 additional individuals for inclusion in the study, 2 of whom we also interviewed, the last potential participant did not respond to the invitation to participate. Notable quotes from across the 15 interviews are displayed in Table 2.

### Barriers to Implementing Sustainability Initiatives

Safety and infection control concerns were the most frequently identified barrier to implementation of sustainability initiatives. This concern was most often brought up by healthcare workers providing direct patient care and more frequently for interventions in the domains of removing or reducing supplies. However, participants from the hospital epidemiology/infection prevention and control department did not share these concerns. They commented that these interventions are feasible “as long as a hospital-approved disinfectant is used and the appropriate contact time is followed” suggesting that interventions to reduce supplies, such as elimination of disposable drapes on surgical tables, are not of concern from an infection prevention perspective.

After infection prevention and control concerns, the three most identified barriers were: 1) outside of expertise (meaning the respondent was unsure), 2) operations/flow/capacity constraints, and 3) knowledge uptake/education. These concerns were widespread throughout the sample of participants and focused mostly on domains of process or policy shifts and reusable supplies. One participant from the Central Sterile Department, which sterilizes reusable surgical instrumentation locally, stated that “a lot of it comes down to training, competency, accessibility, and inclusion of the staff on the process … if the staff aren’t included in the proper setup and also the why of what we’re doing … things are discarded inappropriately simply because someone didn’t know or they didn’t have access to what they needed” regarding staff members compliance and buy-in of sustainability initiatives.

The least frequently mentioned barriers were those relating to financial cost, data needs, burden on staff, consensus building/approval, labor cost, and regulations. Financial cost was most often mentioned by nurse participants, followed by administrative participants, and was specifically a concern when asked about carbon offsets and reusable supplies. Paucity of data was primarily seen as a barrier to implementation of interventions related to supply reduction and reusable supplies, specifically when related to the safety and efficacy of the proposed intervention.

### Data Needs for Implementation of Sustainability Initiatives

Overall, participants consistently identified a higher need for *quantitative* data for sustainability initiatives, particularly in supply-related Domains A and B. The most commonly identified need was for safety and efficacy data, followed by supply cost data. Together, the need for these two subsets of data related to sustainability initiatives made up about half of the total identified data needs. The sustainability coordinator and financial department interviewees, both of whom work at a health system level, most often cited data needs in nearly all intervention categories. Nurses similarly followed this pattern of data need identification; however, surgeons did not. The most heavily requested qualitative data was for observational studies, for example supply use patterns, staff opinions, and a survey of practices in other health systems.

The least frequently identified data needs included volume and waste related to these interventions, regulation and legal support/protections around these interventions, and, lastly, system infrastructure and capacity to accommodate these interventions. Of note, the question on data needs for these interventions received the fewest responses from participants compared to other questions.

### Groups to Engage in Implementation

For each intervention, participants were also asked which groups, within or outside of the healthcare system, they perceived as necessary for the implementation of these sustainability interventions. Internally to the ophthalmology department, participants most frequently mentioned infection prevention and control and clinical operating room staff, such as nurses and scrubs techs, followed by surgeons and clinical managers. Infection prevention and control was seen as a key stakeholder to engage in sustainability initiatives by participants providing direct patient care as well as logistical administrators, such as those working in operations. The same participant groups and senior-level system administrators identified clinical operating room staff as a key stakeholder group in these interventions. When the interventions were related to supply reduction, reusable supplies, and infection prevention and control, clinical operating room staff, surgeons, and clinical management were most frequently identified as key groups. Infection prevention and control was also seen as a key group in implementation of multi-dosing pharmaceuticals.

Of note, participants from the infection prevention and control department did not frequently identify themselves as needing to play a role in the implementation of these sustainability initiatives and instead identified operating room staff, surgeons and clinical managers as key groups to engage. In contrast, operating room staff, surgeons and management most frequently identified themselves as the group needed to be involved in the implementation of sustainability initiatives.

Additionally, procurement and waste management groups were seen as key stakeholders for interventions related to reduction of supplies and reusable supplies. The intervention involving reusable basins and trays received the highest amount of attention as needing both infection prevention and control and waste management intervention.

The remaining groups, including regulatory/legal, departmental administration, pharmacy, system administration, finance, clinical engineering, education/training, external contractors (like waste haulers), patient groups, group purchasing organizations, external stakeholders/peers (like other hospitals), and government were comparably less frequently identified as needing to be engaged in these interventions.

### Steps to Successfully Implement Sustainability Initiatives

When participants were asked about what steps they would recommend to implement these sustainability interventions, participants most commonly identified a bottom-up or consensus-building approach. Nursing staff recommended a bottom-up approach more than any other group of participants interviewed. One participant outlined the benefit of a bottom-up approach:

You always want to get the senior ophthalmologist to present [the proposed sustainability intervention] to the group, whether it’s the chair of the department or one of the senior folks. That always carries much more weight than when it comes from a senior administrator coming in and saying ‘Hey, change this!’ To get one of them to be behind it and support, goes much further because it’s a peer-to-peer type thing.

This approach was most often mentioned in response to questions related to interventions that reduce or reuse supplies. Additionally, participants who mentioned taking a bottom-up approach often suggested that surgeons take the lead on implementing this change.

Staff education and training was the next most-cited step needed to implement these sustainability initiatives. This approach was most frequently recommended by participants from the finance department, followed by nursing staff participants. Education and training were most often recommended as steps for implementation of interventions for waste sorting practices, as well as introduction of reusable supplies. Of note, participants from the infection prevention and control department only mentioned trial/pilot testing and education and training as potential steps to implementation.

Surgeons had an even mix of recommendations, including the bottom-up/consensus building approach, trialing/pilot testing, education and training, and local top-down approaches (such as internal policy setting). No interviewees suggested an external top-down approach, such as state or federal regulatory shifts.

### Perceived Benefits of Sustainability Initiatives

Participants were not asked specifically about perceived benefits of the proposed sustainability interventions, yet many participants cited the benefit of interventions during their interviews. The most frequently identified benefit that emerged across various interviews was that of hospital waste reduction. This benefit was identified most frequently when the intervention involved custom pack supply optimization and multi dose pharmaceuticals, and it was most frequently identified by sustainability administrators and nurses. Additionally, operations, flow, and capacity improvements emerged as perceived benefits, specifically related to multi-dose pharmaceuticals and hub-and-spoke operating room scheduling. This benefit was most frequently identified by participants who provide direct patient care, such as nurses and surgeons. Financial gains, employee engagement and retention, and patient interest were also mentioned as perceived benefits, although not as frequently as the aforementioned benefits. Financial gains were most frequently associated with reduction of supply and introduction of reusable supplies interventions. Employee engagement and retention and patient interest were almost exclusively associated with carbon offset interventions.

### Follow up Interview with Infection Prevention and Control

After conducting all interviews and completing coding of all responses, researchers decided to conduct a follow-up interview with an infection prevention and control representative to talk about the results. During this interview the representative clarified the role of the infection prevention and control department as a “consult resource” for people within the health system to ask about infection prevention policies, to propose changes to these policies, and to bring actions to their attention that may be of infection prevention concern. However, the representative stated that often “infection control’s involvement is inappropriate,” citing that the department may be consulted on matters that are already outlined in their policies, and while these policies are available to all staff members of the health system, they are “accessible on a portal that is neither reliable nor easily navigable.”

During the interview, the infection prevention and control representative worked through the example of switching from a full body drape to a face drape for ophthalmologic surgeries, to illustrate how they envision the implementation of this sustainability initiative. Of note, they mentioned that this intervention would not have to go through an infection prevention and control audit, because the department’s primary concern is that sterile procedures continue to be followed despite drape choice. The representative also suggested that staff interested in implementing this change reach out to logistical partners, such as supply chain or procurement departments, to understand the feasibility of this intervention.

During this interview, there was also mention of the need for someone to take on the primary role of driving the implementation of these initiatives from the ground up. The participant also commented on the discrepancy in concern for infection control and safety between nurses and surgeons, stating that the former group may be more hesitant to take on initiatives that have a perceived increased infection risk because of structural and hierarchical concerns. Additionally, the infection prevention and control representative noted that ultimately pursuing changes through the health system’s medical board would provide a definitive decision on the implementation of sustainability interventions.

## Discussion

As the climate crisis escalates, healthcare systems must begin to critically evaluate their role in perpetuating this crisis and take steps to combat it. This study identifies specific challenges, needs, and opportunities for implementing sustainability initiatives in the ophthalmologic setting. Themes of central importance to implementation of sustainability initiatives from interviewed healthcare professionals include the disconnect between policy and practice, and the key role of bottom-up change as a powerful avenue for implementation.

### Policy versus Practice

Concern for infection prevention and patient safety was the primary perceived barrier by a wide variety of interviewed healthcare professionals. However, both of the interviewed infection prevention professionals clearly expressed their comfort with many of the proposed interventions, as long as they complied with pre-established infection prevention and safety policies. The juxtaposition of these two perspectives, from the people who write the policies and the people whose practice is dictated by these policies, illuminates a dichotomy between policy and practice.

System size and resulting bureaucracy play roles in preventing change in the healthcare system. The structure of a modern academic health center is vast and heterogenous, often reflecting the system’s unique health, financial, and educational missions. These structures work to take on a large volume of patient care, ensure financial prosperity, and train future healthcare workers, creating a system in which there are many layers of governance, all producing innumerable policies and procedures to advance their respective goals. For the people providing direct patient care, this means their work in caring for people is influenced by a massive network of policies made by people with varying levels of connection to this work.

Given this structure of healthcare management, it is not surprising that policy and practice of healthcare are disconnected. However, the effects of a convoluted bureaucratic structure are not unique to healthcare. A systematic review of barriers to innovation in the public sector found that organizational structure affects not only the way change and innovation are implemented but also how they are conceived and adopted (28). As healthcare systems become more consolidated and administration becomes more expansive it is imperative that system leadership remain connected to front line workers and patients.

The intervention of removing disposable covers on the patient bed and draping on surgical tables drew many concerns from clinical staff. Seven out of 15 interviewees, including all nurses and one surgeon, thought this intervention might draw safety and infection prevention concerns. However, the infection prevention and control officer stated that they had “really [no concerns], not from an infection control perspective, because wiping with an approved cleaner would be considered equivalent. And it’s even in our policy that’s equivalent.” Despite hospital policy that enables more resource-efficient care, clinical practices still default to a more wasteful, single-use supply first approach. Clinical staff remain unaware that hospital policies allow for implementation of this intervention with relative ease, and a question remains about how best to either inform staff of the policy or encourage adoption of the equivalent-sterility, no-draping method system-wide, so that all care (not just cataract surgeries) defaults to a more resource-efficient approach.

Other interventions may require more support from other stakeholders, but similarly did not pose an infection risk from that officer’s perspective. Reusable surgical gowns are “a practice that used to be done frequently. And this would require consultation with the building service because it requires a very specific vendor or system to be put in place. As well as central sterile, as well as supply chain, because they’d have to purchase and maintain a stock of these. They actually already do have them, by the way, just in case we ever run out of the other ones.”

Connection between administration and frontline workers is of utmost importance if change is to be successful. In a study examining the characteristics of successful change in healthcare, change was perceived as successful when healthcare workers have influence over the change, are able to prepare themselves for change, and align with the value and purpose of the change (29). In giving front line workers a central role in the implementation of change and innovation, healthcare systems can begin to tackle interdisciplinary challenges such as the climate crisis.

### Bottom-Up Change

Another theme that emerged across interviews was that these interventions would best succeed if they were started and led by front line healthcare workers. Participants from all backgrounds, including those involved in providing direct patient care themselves, consistently cited that implementation would be most effective when it came from surgeons and nurses. Some participants specifically mentioned that change would be more effective when coming from the bottom-up because workers would be able to build consensus and foster buy-in of the intervention from their peers. However, this theme in the context of the health system presents a nuanced challenge: who will take ownership of these changes.

Participants throughout the study described change from the bottom-up as happening when there is a person who champions the idea and takes on a leadership role in developing, building support for, and coordinating the implementation of an idea. This method of implementation is particularly effective because change emerges from the identified needs of a community and is brought to life by the community. However, in healthcare systems, emergent change from the bottom-up is more complex, particularly given the high rates of physician and nurse burnout and the breadth of specialties and departments where champions would be needed. Physicians increasingly report feeling more emotionally exhausted due to overextension, more depersonalized from their work as a consequence and report a low level of feeling personally accomplished. Physician burnout has also been associated with increased medical error and decreased quality of care, contextualizing the challenge of taking on systems level quality improvement initiatives, such as sustainability initiatives, as a healthcare worker providing direct patient care (30). As champions of sustainability in the healthcare system look to achieve their goals, health systems need to build broad coalitions of leaders and community members who can share the logistical responsibilities of change implementation, providing a promising bottom-up centered approach to implementation. Health systems committed to climate action should also create incentives to encourage these bottom-up champions, including financial incentives, tying promotion criteria to engagement in sustainability activities, and offering protected time for developing, testing, garnering support, and implementing interventions.

The healthcare profession has a deep-rooted history of hierarchy, which typically places the people with the highest levels of education in the positions of most power (31). This preferentially places physicians at the top of this power structure, a profession that until recently was not accessible to many diverse people from various racial/ethnic, gender, sexual orientation, socioeconomic status, physical ability, and immigration backgrounds (32). This creates a power structure that poses a significant barrier for allied health workers, nurses, physician assistants, pharmacists, and other members of the healthcare team, who may be trying to advocate for and implement change in the health system (33). Acknowledging the challenges that this hierarchy creates to implementing change is the first step healthcare systems can take to begin fostering an environment where all members of the healthcare team feel empowered to improve our system. All system stakeholder perspectives should be valued, because different roles see different challenges and potential improvements for the larger system.

Additionally, change from the bottom-up in healthcare must also work to integrate the perspectives and experiences of the communities being cared for. Participants in this study did not mention the role that patient groups could play in the implementation of sustainability initiatives, yet integrating patient perspective into health system policies and initiatives presents a powerful opportunity to dismantle the healthcare hierarchy and build a patient centered system (34). Despite the various challenges that bottom-up implementation faces in the healthcare setting, it also presents a valuable opportunity to combat the climate crisis by integrating the perspectives of people who experience adverse climate related health outcomes.

Any system stakeholder attempting to enact sustainability interventions must be properly supported by healthcare leaders. Broadly implementing appropriate sustainability and resource-efficient practices in a health system is bound to be complicated, due to the sheer number of potential stakeholders. Without proper knowledge of the network of stakeholders and incentives for acting, individual stakeholders are unlikely to either engage in sustainability change management or enact sustainability initiatives, particularly if they are in roles with historically little power. Without administrative, financial, and managerial support for sustainability interventions, engaged individual stakeholders may face greater issues with backlash or retaliation. For example, if a nurse in this health system wanted to switch to face drapes rather than full body drapes, their clinical colleagues may be unsupportive, claiming infection prevention concerns. In a more organized effort, this nurse would be connected to the infection prevention and control department who might inform them that “for something like ophthalmologic surgery, where it’s very contained and controlled, a smaller sterile field from my perspective wouldn’t be a risk.” In short, departmental champions must receive support and incentives from higher up in the health system. They cannot act in isolation.

### Limitations of our study

This qualitative study was conducted at a single, large academic medical center. Though the ophthalmology department operates largely out of one ASC, they are beholden to many rules and regulations descending from a much larger health system. The financial, leadership, and systems structure here will differ from other places where cataract surgery is conducted, even within the US. In places where physicians are also owners of the medical facility, stakeholder dynamics and power structures will differ substantially. Complexity of systems will vary, leading to different conclusions, particularly in relation to enacting existing health system policies. We also chose to question stakeholders on interventions specific to this institution. Other health systems may already be engaging in these interventions and may need to vet the barriers and facilitators to other sustainability interventions.

We did not sample with the goal of reaching theoretical saturation, instead interviewing everyone in the health system who responded to our requests. Though many of the interviewee responses were similar, it is possible other system stakeholders we did not interview could have different opinions. We did not interview potential stakeholders outside of our health system, such as upstream suppliers, downstream waste haulers, or representatives from accrediting bodies or regulatory agencies.

## Conclusions

This qualitative study is the first of its kind to assess a variety of health system stakeholder perspectives on implementing sustainability interventions in cataract surgery. Environmental sustainability and resource efficiency are increasingly popular topics for healthcare providers. Successfully implementing appropriate sustainability initiatives in a health system requires the engagement of many system stakeholders. Our study shows that system stakeholders are aware of the potential barriers to sustainability implementation from other stakeholder groups but may not be aware of the ways in which those stakeholders already support interventions. A significant example was the concern front-line clinicians placed on the safety or infection prevention concerns of suggested interventions, compared to the response of the infection prevention and control stakeholder, who suggested that most of the interventions were already compliant with existing health system policies. Clearer communication or enforcement of more resource-efficient policies needs to be achieved.

In addition, most stakeholders thought that education and training could be improved to better implement sustainability interventions, and that a bottom-up model utilizing “sustainability champions” might prove the most effective pathway toward accomplishing sustainability interventions for the health system. Health systems should, therefore, encourage the growth of the bottom-up model by offering administrative, financial, and other support for departmental champions, perhaps even creating a network of crucial stakeholders to validate and encourage any changes.

## Figures and Tables

**Table 1:** Interview Guide Framework

Topic		Question Set
*Domain A*	*Removing or Changing Supplies*	*a. Do you have any concerns with this intervention?* *b. Which data, if any, would be needed to validate this intervention?* *c. Which groups or individuals would need to be consulted to enact this intervention?* *d. What steps would you recommend to implement this at [case location]?*
*Intervention 1*	*Reduce supplies in disposable custom pack*	
*Intervention 2*	*Use a face drape instead of full-body drape*	
*Intervention 3*	*Remove disposable covers on patient bed and surgical tables*	
*Intervention 4*	*Patients wear own clothing during surgery*	
**Domain B**	**Reusable supplies**	*Same as above*
*Intervention 1*	*Replace single use disposable patient restraints with reusable*	
*Intervention 2*	*Replace single use disposable plastic basins/trays with reusable*	
*Intervention 3*	*Replace single use disposable sterile surgical gowns with multiple use sterile surgical gowns*	
**Domain C**	**Pharmaceuticals**	*Same as above*
*Intervention 1*	*Multi-dose eye drops in pre-operative area*	
*Intervention 2*	*Multi-dose eye drops in the OR*	
**Domain D**	**Process or policy shifts**	
*Intervention 1*	*Utilize a hub-and-spoke model, where surgeon alternates between two ORs*	*a. ...could this method be utilized more frequently? Why or why not?* *b. Which groups or individuals from [case location] would need to be consulted to enact this intervention more frequently?*
*Intervention 2*	*Improve waste sorting practices*	*a. ...what are some approaches that [case location] can use to increase proper sorting of waste?* *b. Which groups or individuals would need to be involved to achieve this intervention?*
*Intervention 3*	*Pay for carbon offsets at the health system level*	*a. ...what would be some barriers to [case location]purchasing carbon offsets for their surgeries and other medical activities?* *b. Which groups or individuals would need to be consulted to enact this intervention?* *c. What steps would you recommend to implement this at [case location]?*
*Intervention 4*	*Pay for carbon offsets at the department level*	

**Table 2:** Exemplary Responses

Result Category	Responses
**Barriers to Implementing Sustainability Initiatives**	● I really am afraid of risking any infection. ● The concerning part would be just maintaining the sterile field and making sure the sterile field is sufficient for the needs of the procedure. ● *For something like ophthalmologic surgery, where it’s very contained and controlled, a smaller sterile field from my perspective wouldn’t be a risk*.^a^ ● I'm not sure if we are risking infection control measures. How effective would it be just to wipe it? ● Just wiping the table doesn’t make it sterile anymore. It’s just clean. I don’t think this could happen. ● *As long as a hospital approved disinfectant is being used and the contact times are followed, which is the time where the surface is kept wet, I wouldn’t have any concerns.*^a^ ● The potential to affect sterility is of utmost concern to the surgeons. I think that we should be as sustainable as we can, but not at the expense of sacrificing patient care. ● The spacing and storage of equipment is a barrier, where we would be able to place the basins and where they would be separated? I also think that’s not going to be environmentally friendly because you're going to have to sterilize them. ● I would be concerned infection control wise, with the amount of liquid that we use here it might end up not being sustainable. You would also need to look at laundry impacts. ● There is evidence across many drugs that the use of multiple dose vials leads to infection or creates the risk of greater infection. There are great outbreaks that are associated with adenovirus, particularly amongst eye drops. ● I think in order to implement this, you need to know how we bill for [multi-dose eye drops], you may be forced to bill a patient for a bottle that they didn’t fully receive. ● I don’t know if we have the space to do this all the time, and the schedule would need to be coordinated well.
**Data Needs for Implementation of Sustainability Initiatives**	● You probably need to actually go into a procedure, see the process, and from there actually quantify everything that is not used. ● We would have to look at infection control outcomes and ensure that these interventions would be safe for patients. ● Well, you need to look at the endophthalmitis rate after cataract surgery and see if it’s any different. ● I think just again any data about the safety of wearing your normal clothes during surgery. ● I would really want to get input from infection control and make sure it would be safe. ● You need to look at the cost [of reusable basins] and what the cleaning and sterilization process would look like in terms of the energy that consumes. ● We would have to look at other facilities and see how it works and also talk with pharmacy, because for multi-dose it would have to prepared it to prevent any type of cross-contamination.
**Groups to Engage in Implementation**	● You would want medical staff to approve it. They are the teams you need to consult because they're the ones who use the supplies daily. ● That would probably be good to run by the clinical staff and infection prevention to ensure sterility. ● Nursing would probably follow up and want education on the safety and quality of the [smaller drape] just to be sure that it’s okay. ● Anytime you're replacing anything that has sterility concerns, you need to know about infection control. ● *From an infection control perspective, we would not need to become involved because wiping would be considered equivalent, and that’s even in our policy that it’s an equivalent*.^a^ ● Central sterile department who, I guess, would validate the storage spaces that we have within our two units, and how they would be able to store and sort these basins ● Pharmacy would need to approve multi-dose pharmaceuticals and of course we would also need buy-in and input from our nursing colleagues and from infection prevention and control, so something like this we would make a collaborative policy.
**Steps to Successfully Implement**	● The tabletop review meeting where nurses or surgical techs who are familiar with the cases can provide input to physicians who are interested in reducing waste to eventually help influence their peers to adopt new practice or use fewer items. ● I think for this you really do have to And a specific physician who would advocate for the face drape and have them come up with their justification to present that to leadership, supply chain and clinical engineering because physicians really are the ones who hold the power over which products are used during an operation. ● Having the chair of the department or one of the senior ophthalmologists communicate why we are making the change. ● You would probably just want to make sure that the OR staff is okay with it. ● *As long as the sterile field is maintained the change would be okay*.^a^ ● *It would just be doing it and getting practice*.^a^ ● I think the education piece is kind of the biggest step, amongst your pharmacy team members who are doing the restocking of these [multi-dose] medications to make sure that they're appropriately labeled and then for the nursing and physician teams to develop understanding of the safety, efficacy and billing process. ● For this we would have to change our charging units to be by drop instead of for the whole bottle. ● Additional staff education would be the first, making recycling procedures clear and setting the staff and OR up for success. ● Placing the appropriate recycle bins in the OR and properly labeling them to let them know that you know this waste goes into this waste bag, doing some education and sharing the impact of the recycling with the team.
**Perceived Benefits of Proposed Sustainability Initiatives**	● Talking to the actual vendors who are making the custom packs to translate what you learned about supply usage into impact that could be applied to hundreds of academic medical centers and hospitals across the country. ● Multi-dose eye drops is more efficient, and it’s less of a waste of product. ● I think that from both the sustainability and environmental impact perspective and also the supply chain perspective being responsible with medication supply and using it as multi-doses is super helpful. ● I would say with carbon offsets, there are also many different creative ways to get offsets. It doesn’t always have to be reforestation or something that we typically think of. There are some offset projects that are community benefit such as upgrading energy efficiency in low-income housing.

a response from the Infection Prevention and Control participants

## Data Availability

The datasets used and analysed during the current study are available from the corresponding author on reasonable request.

## Abbreviations

GHGs: Green House Gases
ASCs: Ambulatory Surgical Centers
AAO: American Association of Ophthalmology
ASCRS: American Society of Cataracts and Refractive Surger
